# Evidence-based guidelines for controlling pH in mammalian live-cell culture systems

**DOI:** 10.1038/s42003-019-0393-7

**Published:** 2019-04-26

**Authors:** Johanna Michl, Kyung Chan Park, Pawel Swietach

**Affiliations:** Department of Physiology, Anatomy and Genetics, University of Oxford, OX1 3PT Oxford, UK

**Keywords:** Cell culture, Cancer microenvironment, Cell biology, Cytological techniques

## Abstract

A fundamental variable in culture medium is its pH, which must be controlled by an appropriately formulated buffering regime, since biological processes are exquisitely sensitive to acid–base chemistry. Although awareness of the importance of pH is fostered early in the training of researchers, there are no consensus guidelines for best practice in managing pH in cell cultures, and reporting standards relating to pH are typically inadequate. Furthermore, many laboratories adopt bespoke approaches to controlling pH, some of which inadvertently produce artefacts that increase noise, compromise reproducibility or lead to the misinterpretation of data. Here, we use real-time measurements of medium pH and intracellular pH under live-cell culture conditions to describe the effects of various buffering regimes, including physiological CO_2_/HCO_3_^−^ and non-volatile buffers (e.g. HEPES). We highlight those cases that result in poor control, non-intuitive outcomes and erroneous inferences. To improve data reproducibility, we propose guidelines for controlling pH in culture systems.

## Abstract (web summary)

Johanna Michl et al. use real-time pH measurements of cell culture media and intracellular pH data from live-cell culture conditions to explore the effects of commonly-used buffers. Based on these data, they propose guidelines for controlling pH in cell culture systems and improving reproducibility.

## Introduction

Biomedical laboratories routinely perform cell culture to produce a cellular environment that is precisely defined, well controlled and physiologically relevant. Among the main chemical variables of culture systems is the concentration of H^+^ ions, oftentimes referred to as protons. These ions are present in every aqueous compartment, not least from the ionization of water. Various solutes can become protonated, thereby establishing multiple chemical equilibria involving H^+^ ions. Consequently, the concentration of free H^+^ ions is not intuitive to predict, but fortuitously simple to measure (e.g. with electrodes or indicator dyes). For over a century, the pH scale has been the reporting standard for the concentration of free H^+^ ions^1^, that is, the form that is able to protonate targets and post-translationally modify proteins, such as enzymes or receptors^2–4^. The much larger pool of buffered H^+^ ions can, however, influence pH through dynamic re-equilibration^5^.

There is still a widely held misconception that buffers have an inherent ability to set the pH of a solution to a pre-defined level. More accurately, in a system of one dominant buffer, pH is related to the concentration of the buffer’s protonated (HB) and unprotonated (B) forms, and the acid dissociation constant (p*K*_a_):

1 $${\mathrm{pH}} = {\mathrm{p}}K_{\mathrm{a}} + {\mathrm{log}}\frac{{\left[ {\mathrm{B}} \right]}}{{\left[ {{\mathrm{HB}}} \right]}}.$$

Consider the dissolution of HEPES buffer (4-(2-hydroxyethyl)-1-piperazineethanesulfonic acid), typically supplied as a powder of the free acid form. This produces an acidic solution that must be titrated (with base, e.g. NaOH) to the desired pH; once the [B]/[HB] ratio is raised to the required level, pH will remain stable, unless there is an additional source of acid or base. In live-cell culture, pH disturbances are an inescapable consequence of metabolism and there is a general tendency for media to undergo acidification, the extent of which is also a function of medium buffering capacity. In a closed system, it can be derived mathematically (see Supplementary Note) and shown empirically^5^ that peak buffering capacity is attained when the buffer’s protonated and unprotonated forms are equimolar, that is, when medium pH aligns with the buffer’s p*K*_a_. Many exogenous buffers are available, covering a wide pH range, including HEPES (p*K*_a_ = 7.3; 37 °C), PIPES (piperazine-*N*,*N*′-bis(2-ethanesulphonic acid); p*K*_a_ = 6.7) and MES (2-(*N*-morpholino)-ethanesulfonic acid; p*K*_a_ = 6.0)^6^.

A more active means of maintaining high buffering capacity involves the regulation of [HB] and [B], so that their ratio is kept at an optimum^5^. This strategy underpins the reason why complex organisms rely on CO_2_/HCO_3_^−^ buffer (despite low p*K*_a_ = 6.15)^7^, and have evolved gas exchange surfaces (e.g. lungs) and ion transport epithelia (e.g. kidneys) to empower CO_2_ and HCO_3_^−^ homeostasis^8^. The combination of CO_2_ (an acidic gas) with HCO_3_^−^ (a base) produces quantitatively the most important buffer in extracellular body fluids. In culture systems, this so-called carbonic buffer is stabilized by adding an amount of HCO_3_^−^ salt to media and enriching the incubator's atmosphere with CO_2_. Here, we relate HCO_3_^−^ and CO_2_ with pH, show how the system responds to changes in its components and demonstrate how the equilibrium is affected by non-volatile buffers (NVBs) added to augment buffering capacity. Furthermore, we explain how certain cell culture manoeuvres may lead to poor pH control. While a number of high-profile guidelines relating to cell culture have been published recently^9–13^, they do not comprehensively cover the aforementioned issues pertaining to pH. Based on our observations, we propose guidelines for good practice in controlling pH in culture systems.

## Results

### Monitoring culture medium pH under incubation

**Fig. 1:**
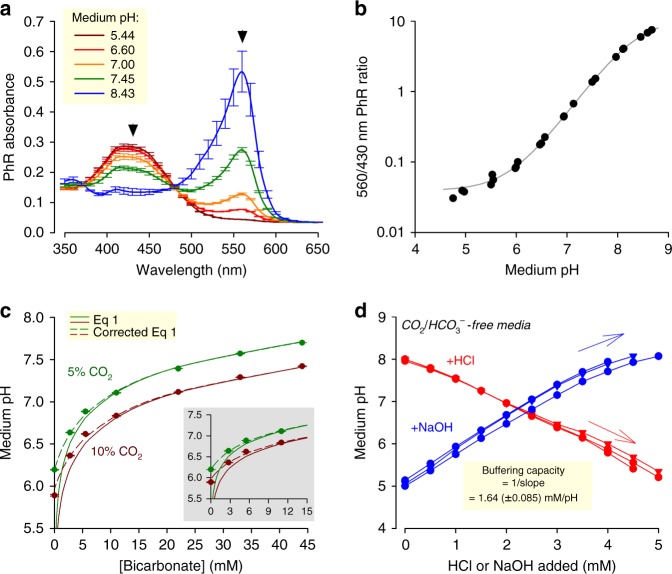
Measuring and setting medium pH under incubation. **a** Absorbance spectrum of Phenol Red (PhR) in Dulbecco’s modified Eagle’s medium (DMEM) (D7777) with 10% foetal bovine serum (FBS), 1% penicillin–streptomycin (PS), 10 mM HEPES (4-(2-hydroxyethyl)-1-piperazineethanesulfonic acid) plus 10 mM 2-(*N*-morpholino)-ethanesulfonic acid (MES), and titrated (5 M HCl or 4 M NaOH) to the indicated pH. Arrows indicate wavelengths for optimal ratiometric analysis. **b** pH dependence of 560/430 nm ratio, fitted to curve: pH = 8.35 + log((10.9 – ratio))/(ratio – 0.0392)). **c** Controlling equilibrium pH by varying pCO_2_ and [HCO_3_^−^] in DMEM (D7777) supplemented with 10% FBS and 1% PS. Dashed line plots Eq. 1. Continuous line is best fit to Eq. 3 (corrected version of Eq. 1), which accounts for buffering by serum (best fit: 1.11 mM pH^−1^). Inset replots the data at low [HCO_3_]. **d** Empirical determination of intrinsic buffering capacity of DMEM (D7777; 10% FBS/1% PS, 25 mM glucose) nominally lacking buffers; titration with either HCl or NaOH. Inverse of slope  provides an estimate of buffering due to serum proteins and media salts. All measurements were repeated three times (three technical replicates each). Data are shown as mean ± SEM

Buffers are included in culture media to control acidity, yet the ensuing pH is not routinely monitored. This becomes a quality control issue whenever the components of buffering are being disturbed: for example, in response to metabolic acid production, or as a consequence of transferring media between atmospheres of different CO_2_ partial pressures (pCO_2_). The dye Phenol Red (PhR) is routinely included in the media to allow investigators to assay medium acidity^14,15^. Such assessment could be done ‘by eye’, but a more quantitative readout of pH is obtainable from the PhR absorbance spectrum, which can be recorded on plate-reader platforms with an incubator chamber (e.g. Cytation 5, BioTek). To obtain a calibration curve, freshly prepared standards of known pH were scanned in a CO_2_-free atmosphere to prevent the acidifying effect of CO_2_. Calibration solutions had no added HCO_3_^−^, as otherwise this basic substance would have reacted slowly with H^+^ ions and then escape as CO_2_ gas. Fig. 1a shows absorbance spectra of PhR in bicarbonate-free Dulbecco’s modified Eagle’s medium (DMEM) (D7777, Sigma-Aldrich) supplemented with 10% foetal bovine serum (FBS) and 1% penicillin–streptomycin (PS; 100 U mL^−1^ penicillin, 0.1 mg mL^−1^ streptomycin), 10 mM HEPES and 10 mM MES (2-(N-morpholino)-ethanesulfonic acid), and titrated to a target pH with NaOH. To correct for pH-independent variables, such as light path or PhR concentration, absorbance was sampled at two wavelengths that respond differently to pH. Good resolving power is attained by rationing absorbance at 560 and 430 nm (Fig. 1b). The best-fit equation can then be used to convert PhR ratio assayed in subsequent experiments to pH.

### Setting medium pH by pCO_2_ and [HCO_3_^−^]

In principle, it is possible to control pH with one of many commercially available buffers, but the most physiologically relevant one is CO_2_/HCO_3_^−^. Incubators maintain a CO_2_-rich atmosphere (typically 5%) to enable CO_2_/HCO_3_^−^ buffering. A salt of HCO_3_^−^ must be included in the  medium to balance the spontaneous H^+^-yielding CO_2_ hydration reaction, and stabilize pH:

2 $${\mathrm{CO}}_{\mathrm{2}}\left( {{\mathrm{gas}}} \right)\rightleftarrows{\mathrm{HCO}}_{\mathrm{3}}^{\mathrm{ - }}\left( {{\mathrm{aq}}} \right){\mathrm{ + H}}^{\mathrm{ + }}\left( {{\mathrm{aq}}} \right).$$

Conveniently, pH can be titrated in the range between ~6 and ~8 by varying the concentration of HCO_3_^−^. Figure 1c plots the relationship between pCO_2_, [HCO_3_^−^] and pH measured in DMEM (D7777, Sigma-Aldrich) containing 10% FBS and 1% PS. To keep osmolality constant, any reduction in NaHCO_3_ was matched by a compensatory rise in NaCl. Over the alkaline range, pH reported by PhR is in very close agreement with the prediction of the Henderson–Hasselbalch equation (Eq. 1), consistent with CO_2_/HCO_3_^−^ being the dominant buffer (p*K*_a_ = 6.15; CO_2_ solubility 0.024 M atm^−1^, i.e. 1.2 mM in 5%)^7^. However, at low [HCO_3_^−^], Eq. 1 underestimates pH. This discrepancy arises because the so-called ‘intrinsic’ buffers in medium (such as proteins included in serum) react with H^+^ ions generated by CO_2_ hydration, pushing the equilibrium (Eq 2) towards a higher [HCO_3_^−^] and lower [H^+^], that is, a less acidic medium. This correction is derived mathematically in the Supplementary Note. Thus, the concentration of HCO_3_^−^ required for attaining a target pH is:

3 $$\left[ {{\mathrm{HCO}}_3^ - } \right] = \left[ {{\mathrm{CO}}_2} \right] \times 10^{{\mathrm{pH}}_{{\mathrm{target}}} - 6.15} + \beta _{\mathrm{intrinsic}} \times \left( {{\mathrm{pH}}_{{\mathrm{target}}} - 7.4} \right).$$

By best fitting the data to this equation, intrinsic buffering (*β*_intrinsic_) was estimated to be 1.1 mM pH^−1^. Alternatively, *β*_intrinsic_ can be measured empirically from the response of pH to the step-wise addition of acid or base (1.6 mM pH^−1^; Fig. 1d).

### Stability of CO_2_/HCO_3_^−^ buffering

**Fig. 2:**
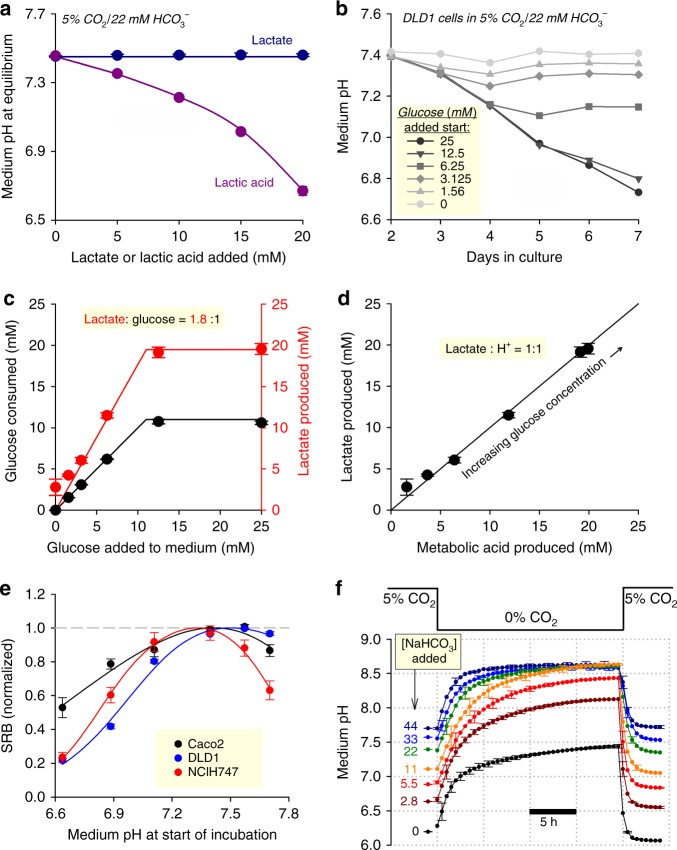
The control and stability of pH in CO_2_/HCO_3_^−^-buffered medium. **a** Effect of increasing [lactic acid] or [lactate] on equilibrium pH of medium (DMEM D5030) with 22 mM NaHCO_3_ (10% foetal bovine serum (FBS), 1% penicillin–streptomycin (PS)) placed in 5% CO_2_. **b** Effect of metabolic lactic acid production by DLD1 cells (seeding density 4,000 cells per well, growth area 0.32 cm^2^ per well) on the pH of medium (DMEM D5030; 10% FBS,  1% PS) containing 0–25 mM glucose (osmotically compensated with NaCl). Error bars omitted for clarity. **c** Effect of varying starting glucose concentration on net glucose uptake and lactate production probed on the seventh day of incubation. 90% of glucose is metabolized to lactate. **d** Relationship between lactate production (measured by biochemical assay) and total acid production (calculated from the pH change and buffering capacity). Slope of 1.0 indicates that medium acidification is due to lactic acid production. **e** Cell growth of three colorectal cancer cell lines (seeding density 4,000 cells per well, growth area 0.32 cm^2^ per well) measured from protein biomass (sulforhodamine B (SRB) assay) after 6 days of incubation in DMEM (D7777; 10% FBS, 1% PS, 25 mM glucose) over a range of starting pH attained by varying [HCO_3_^−^] at constant 5% CO_2_. Data are normalized to the optimum pH derived by best fit to biphasic curve. Optimal growth is near the physiological pH of 7.4. **f** Effect of varying pCO_2_ on medium pH, mimicking the withdrawal of medium from under CO_2_ incubation. All experiments were repeated three times (three technical replicates each). Data are shown as mean ± SEM

In most instances, media are prepared to a neutral or alkaline pH, and over this pH range, the Henderson–Hasselbalch equation (1) is adequate for predicting equilibrium pH. However, the robustness of Eq. 1 depends on the accuracy of pCO_2_ and [HCO_3_^−^] measurements. In many instances, it may be appropriate to assume that the amount of HCO_3_^−^ salt added to a medium accurately predicts the final concentration of base; however, some formulations contain weak acids that react with HCO_3_^−^ salts. Under these circumstances, Eq. 1 will underestimate pH, and therefore direct pH measurements are advocated. For example, the addition of 22 mM of NaHCO_3_ to media supplemented with lactic acid will not produce the expected pH of 7.4 due to the titration reaction (Fig. 2a). If, instead, media contained a salt of lactic acid (e.g. Na-lactate), then the acid-titration reaction with HCO_3_^−^ will not take place, and Eq. 1 adequately approximates pH (Fig. 2a). The difference in behaviour between lactic acid and its conjugate base can be explained in terms of equilibria:

$${\mathrm{Lactic}}\,{\mathrm{acid\rightleftarrows Lactate + H}}^{\mathrm{ + }}.$$

Around neutral pH, this equilibrium is shifted far to the right. After dissolving a lactate salt, only a tiny fraction of lactate will protonate, thus the change in pH is negligible. In contrast, lactic acid added to a medium undergoes near-complete deprotonation, which reduces pH.

Physiologically, a principal source of lactic acid is glycolytic metabolism (~1:1 lactate:H^+^ stoichiometry^16^), which inadvertently reduces the pH of a finite volume of medium by reacting with its HCO_3_^−^ ions. An exemplar time course of medium acidification produced by DLD1 cells is shown in Fig. 2b for a range of starting glucose concentrations. Below a starting glucose concentration of ~12 mM, substrate availability is rate-limiting for lactic acid output, measured from lactate accumulation and glucose consumption after 6 days of incubation (Fig. 2c). When glucose availability was not rate-limiting (>12 mM), DLD1 cells were able to produce ~20 mM of lactic acid over a period of 6 days. The extent to which lactic acid production underpins medium acidification was determined by comparing lactate measurements with cumulative acid production:

$${\mathrm{Acid}}\,{\mathrm{produced}} = - \mathop {\sum }\nolimits( \beta \cdot \Delta {\mathrm{pH}}).$$

Here, buffering capacity (*β*) is the sum of intrinsic buffering and CO_2_/HCO_3_^−^-dependent buffering (see Supplementary Note). Plotting the relationship between these two independent measurements (Fig. 2d) demonstrates that in DLD1 cells, medium acidification is entirely accounted for by glycolytic lactic acid production.

Considering the magnitude of lactic acid production, medium [HCO_3_^−^] will invariably fall below starting levels during extended periods of incubation. As a pre-emptive measure, many types of media are formulated to contain 44 mM NaHCO_3_, an excess to provide a safety margin for adequate base. However, at 5% CO_2_, such media will equilibrate at pH 7.7, which is a supra-physiological level that can have untoward effects on cells^6^. To demonstrate the importance of physiological [HCO_3_^−^], cell growth was studied at various levels of pH attained by varying [HCO_3_^−^], over a period of 6 days in serum-containing medium. Cellular growth, in the presence of 10% FBS and 1% PS, was interrogated by a cytotoxicity assay based on the protein-binding probe sulforhodamine B (SRB)^17^. In three colorectal cancer cell lines (NCI-H747, DLD1, Caco2), growth was optimal near pH 7.4, and the effect of incubation at pH 7.7 varied, with the strongest inhibition of growth in NCI-H747 cells (Fig. 2e). The notion of an optimal pH for cell growth has been noted by others^18^, but the molecular mechanism behind this response is not well defined. Reduced proliferation at pH >7.4 may, for instance, relate to excessive debinding of H^+^ ions from sensors and ‘ionic trapping’ of lactate in alkaline cytoplasm.

Another factor that may contribute towards pH instability relates to pCO_2_. In vivo, most mammalian cells will be exposed to a tightly regulated pCO_2_, which helps maintain pH homeostasis. By analogy, feedback circuits in incubators are designed to keep pCO_2_ constant. This environmental constancy is, however, not always possible with cultured cells, as various protocol steps may require transfers between atmospheres of different pCO_2_ (e.g. in and out of an incubator). There are two implications of this. First, a medium that had been titrated in a CO_2_-free atmosphere (e.g. at the bench) will acidify upon placement in a CO_2_ incubator^6^. Second, data collected from cells that had been withdrawn from an incubator may be influenced by the abrupt rise in pH^6^. This issue could be addressed by minimizing CO_2_ loss from the medium (e.g. by enclosing the culture dish in a regulated atmosphere), or by superfusing with solutions pre-equilibrated with 5% CO_2_. The loss of CO_2_ from a medium can be tracked using the time course of alkalinization evoked when HCO_3_^−^-containing media equilibrated at 5% CO_2_ is transferred into a CO_2_-free atmosphere (Fig. 2f). The small volume of media contained in 96-well plates begin to alkalinize immediately, with a time constant of 2–3 h. The reverse reaction has a time-constant of 45 min, indicating that freshly prepared media may require an hour to equilibrate inside a CO_2_ incubator.

### Effect of NVBs on the stability of medium pH

**Fig. 3:**
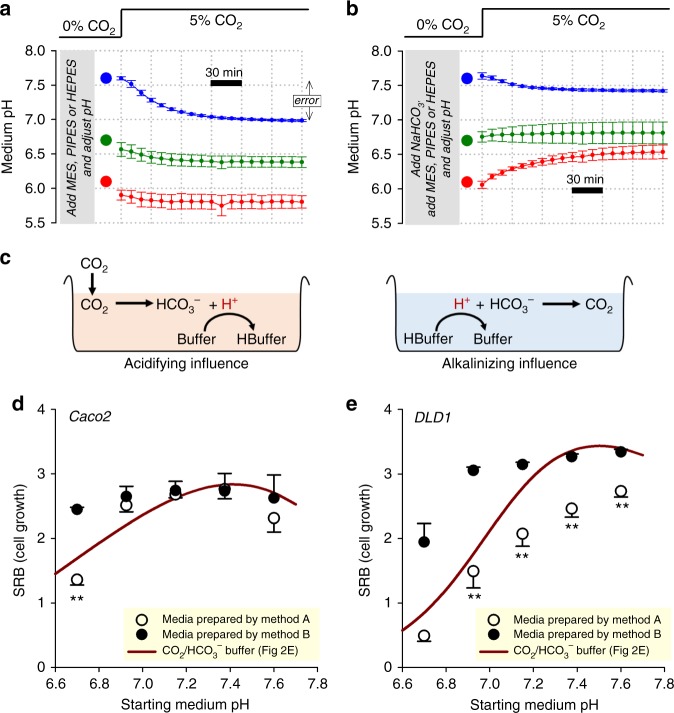
pH dynamics in media prepared with non-volatile buffers without consideration of the CO_2_-HCO_3_^−^ equilibrium. **a** Medium  (DMEM D7777) supplemented with non-volatile buffer (HEPES, PIPES or MES; 20 mM), 10% FBS, 1% PS, and titrated to indicated target pH (large circles). Time courses show pH dynamics (measured from PhR ratio) evoked by placing media inside 5% CO_2_ incubator, showing a tendency to acidify. **b**  Medium (DMEM D7777) supplemented with 22 mM NaHCO_3_ plus non-volatile buffer (HEPES, PIPES or MES; 20 mM), and titrated to indicated pH before placement in 5% CO_2_. Time courses show pH dynamics evoked by placing media in 5% CO_2_ incubator, demonstrating pH instability. **c** Schematic of the chemical processes that underpin medium pH drifts. **d** pH-dependence of Caco2 cell growth (seeding density 4000 cells per well, growth area 0.32 cm^2^ per well) measured from protein biomass (SRB assay) after 6 days of incubation in D7777 (25 mM glucose). Media were prepared by method A (20 mM HEPES/PIPES) or method B (20 mM HEPES/PIPES plus 22 mM NaHCO_3_), and placed in 5% CO_2_. To obtain a range of pH values, HEPES- and PIPES-buffered media were mixed in various ratios. Data are plotted as a function of assumed pH (titrated ‘at the bench’). Results compared against curve obtained with CO_2_/HCO_3_^−^ buffer, plotted against measured equilibrium pH (data from Fig. 2e). **e** Experiment performed on DLD1 cells, a more pH-sensitive line. Data are shown as mean ± SD (**a**, **b**) or mean ± SEM (**d**, **e**). All measurements were repeated three times (three technical replicates each). Statistical tests: two-sided *t* test (**P < 0.01)

The perceived drawbacks of CO_2_/HCO_3_^−^, namely its volatility and weaker buffering at low pH, have led to the use of exogenous NVBs such as HEPES, PIPES and MES^19^. It is crucial that the preparation of media containing such buffers carefully considers the CO_2_/HCO_3_^−^ equilibrium, which takes place under CO_2_ incubation. NVB-buffered medium titrated ‘at the bench’ to a target pH will invariably become more acidic upon placement in a 5% CO_2_ incubator. For example, bicarbonate-free DMEM buffered with 20 mM HEPES (a widely used formulation) acidifies by over half a pH unit upon exposure to 5% CO_2_ (Fig. 3a). Acidification was less pronounced at low pH because the concentration of HCO_3_^−^ required to meet the equilibrium condition is lower. The extent of CO_2_ hydration could be curtailed by supplementing media with HCO_3_^−^ salts. This, however, produces a two-buffer system in which pH dynamics are less intuitive to predict. To demonstrate this instability, media were prepared with 22 mM NaHCO_3_ and one of either HEPES, PIPES or MES. These mixtures were titrated ‘at the bench’ to near the p*K*_a_ of the constituent NVB, and then promptly placed in a 5% CO_2_ incubator for continuous pH monitoring. Media prepared this way demonstrated a substantial degree of pH instability (Fig. 3b). The direction of pH drift is determined by two opposing chemical reactions (Fig. 3c): (i) the acidifying effect of atmospheric CO_2_ dissolving and reacting with the medium; and (ii) the alkalinizing effect arising from the equilibration between HCO_3_^−^ ions and the NVB, a slow process that had started prior to incubation in 5% CO_2_. The balance between these opposing processes depends on the starting pH, and produces an array of responses that are not intuitive to predict (Fig. 3b).

Poorly controlled pH, such as in the instances described above, will impinge on the accuracy and reliability of biological recordings. To illustrate this problem, the pH dependence of growth under 5% CO_2_ incubation was measured in NVB-buffered media prepared either with or without the addition of 22 mM NaHCO_3_, according to the schemes shown in Fig.  3a, b, respectively. Experiments were performed on Caco2 (Fig. 3d) and DLD1 cells (Fig. 3e), representing a weakly and strongly pH-sensitive line, respectively (see Fig. 2e). Growth, measured by the SRB assay after 6 days of culture, was plotted against the pH to which media were titrated ‘at the bench’ (i.e. the assumed pH). In line with previous studies^20^, PIPES was selected for acidic media and HEPES was chosen for alkaline media; to obtain a range of pH, these media were mixed in various ratios. As a control, growth was measured separately in media prepared with CO_2_/HCO_3_^−^ in a manner that produces a predictable starting pH (Fig. 1c). If the error associated with pH instability under CO_2_ incubation were negligible, then the pH dependence of growth measured in NVB-buffered media would be the same, irrespective of the method of medium preparation. However, the measured pH–growth relationship was apparently different, depending on how the medium was prepared. NVB-buffered media prepared without NaHCO_3_ (Fig. 3a; method A) yielded an apparently steeper pH dependence of growth, compared to NVB-buffered media prepared with NaHCO_3_ (method B). This disparity, which was more pronounced in the strongly pH-sensitive DLD1 line, cannot be explained merely by the chemical presence of NVBs because both methods used matching concentrations of HEPES and/or PIPES. Also, the differences do not relate to inadequate [HCO_3_^−^] (e.g. for supplying pH-regulating proteins) because all media inside CO_2_ incubators eventually accumulate HCO_3_^−^ from the spontaneous hydration of CO_2_. Instead, the apparent shifts in pH–growth relationship relate to the pH error incurred during medium preparation. When placed inside a CO_2_ incubator, NVB-buffered media prepared according to method A (Fig. 3a) will undergo an acid-shift across the pH range. This has the effect of over-estimating the degree of growth inhibition at low pH. In contrast, the pH of NVB-buffered media prepared with NaHCO_3_ and titrated ‘at the bench’ (Fig. 3b) will converge towards pH ~7 during CO_2_ incubation. This produces apparently pH-insensitive growth, because the test range of pH is, in reality, narrowed. Ultimately, the error was introduced because medium pH was set in a manner that did not take into account the CO_2_-HCO_3_^−^ equilibrium.

**Fig. 4:**
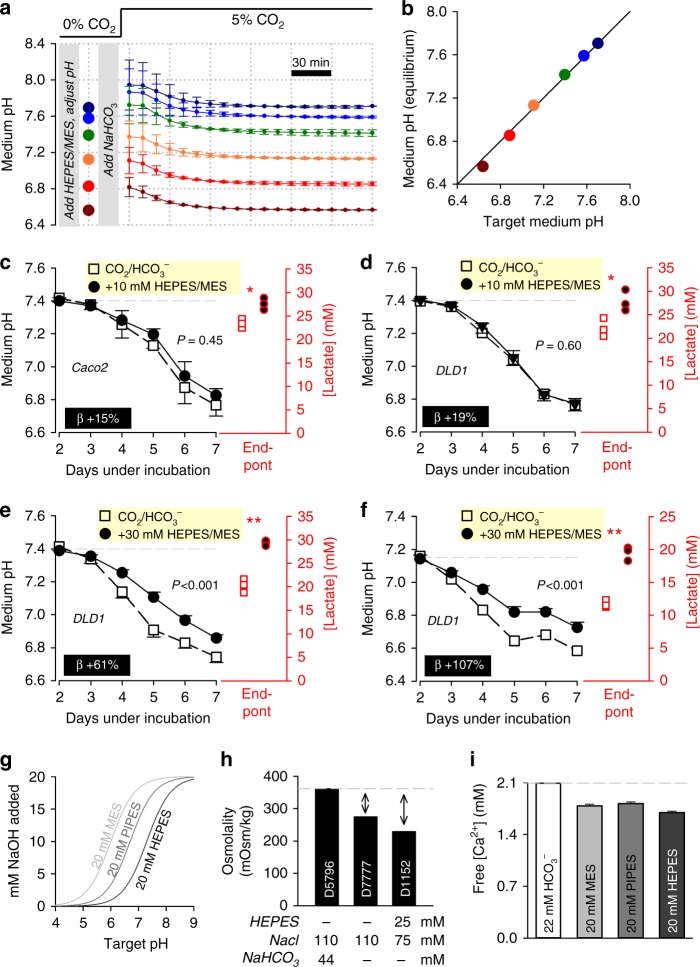
Enhancing buffering capacity of CO_2_/HCO_3_^−^-containing media with non-volatile buffers, with consideration of the CO_2_-HCO_3_^−^ equilibrium. **a** Medium (DMEMD 7777, 10% FBS, 1% PS, 25 mM glucose) supplemented with non-volatile buffer HEPES and MES (10 mM), and titrated to indicated target pH (large circles). NaHCO_3_ then added to a concentration expected to be in equilibrium with 5% CO_2_ at target pH (Fig. 1c). Time course of pH equilibration under 5% CO_2_ from different starting levels. Repeated  three times (three technical replicates each). **b** Good agreement between target and measured equilibrium pH. **c** Time course of medium acidification in Caco2 cells (seeding density 4,000 cells per well, growth area of 0.32 cm^2^ per well). Media buffered with 5% CO_2_/22 mM HCO_3_^−^, or the combination of CO_2_/HCO_3_^−^ plus 10 mM HEPES/MES (a 19% increase in time-averaged buffering, *β*). Medium lactate accumulation at the end point was greater with enhanced buffering. **d** Experiment performed on DLD1 cells. **e** Experiment performed on DLD1 cells with 30 mM HEPES/MES. **f** Experiment repeated from a more acidic starting pH, at which 30 mM HEPES/MES is expected to provide half of total buffering. Measurements repeated four times (three technical replicates each). Statistical tests: lactate measurements tested by two-sided *t* test (*P < 0.05, **P < 0.01); time courses tested by two-way analysis of variance (ANOVA) (*P* value for the effect of buffering is stated). **g** Increasing buffering capacity with non-volatile buffers also increases osmolarity due to the buffer molecules and the base required for titration. Calculated [NaOH] required to titrate MES, PIPES or HEPES buffer to a target pH. **h** Osmolality of three different media formulations. Arrows show gap in osmolality in HCO_3_^−^-free media, which can be filled with buffer and acid–base required for titration, plus additional NaCl required to bring osmolality to a physiological level. Note, in the case of medium D7777 and D1152, a total of 88 and 132 mOsm kg^−1^ can be added, respectively. **i** Free [Ca^2+^] measured by electrode, showing partial Ca^2+^ chelation by non-volatile buffers. Data are shown as mean ± SEM. Repeated three times

Notwithstanding the issues described above, there may be valid reasons to supplement media with NVBs (e.g. to limit medium acidification under long-term cell culture of highly glycolytic cell lines)^20^. The apparent instability of systems containing a NVB plus CO_2_/HCO_3_^−^ (described in Fig. 3) could be addressed by modifying the protocol for preparing media. In the first step, the NVB should be added to bicarbonate-free media, and then titrated to a target level ‘at the bench’. To include CO_2_/HCO_3_^−^ buffer, its components must be added at a concentration ratio that will be in equilibrium with the target pH (Fig. 1c). In the case of HEPES-buffered media titrated to pH 7.4, this would require the addition of 22 mM of NaHCO_3_ and placement in 5% CO_2_. Media prepared this way will equilibrate to the target pH within 2 h when aliquoted into 96-well plates (Fig. 4a). Note that the equilibration takes longer than in the experiment shown in Fig. 2f because of the resistive action of NVBs towards pH changes. Hence, it is possible to combine an exogenous buffer with physiological CO_2_/HCO_3_^−^ to improve overall buffering capacity, and still attain a predictable pH (Fig. 4b).

To test how additional buffering affects the time course of medium acidification, Caco2 or DLD1 cells were cultured in CO_2_/HCO_3_^−^-buffered DMEM supplemented with 10 mM HEPES and 10 mM MES, prepared as described in Fig. 4a. This combination of two NVBs adds a constant buffering capacity over the pH range 6 to 8, making it easier to identify biological effects in media undergoing acidification. Somewhat paradoxically, 10 mM HEPES/MES did not meaningfully reduce medium acidification (Fig. 4c, d), despite the obvious increase in buffering power. However, the enhanced buffering was found to increase lactate production, implying a greater collective glycolytic rate. The resulting pH time course was unaffected, because the augmented buffering capacity was cancelled out by the stimulated metabolic acid production, which can be expressed mathematically as:

$${\mathrm{Change}}\,{\mathrm{in}}\,{\mathrm{medium}}\,{\mathrm{pH}} = - \frac{{{\mathrm{lactic}}\,{\mathrm{acid}}\,{\mathrm{production}}}}{{{\mathrm{buffering}}\,{\mathrm{capacity}}}}.$$

These observations can be explained in terms of the inhibitory feedback of acidity on metabolic rate^21–23^. Augmented buffering reduces the degree of medium acidification, which is permissive for a higher metabolic rate. As expected from a simple pH-operated feedback circuit, the ensuing pH time course will follow an unchanged trajectory. When the experiment was repeated on DLD1 cells using a much higher (30 mM) concentration of HEPES/MES, lactate production was still stimulated, but not to a degree that would offset the increase in buffering (Fig. 4e). The effects of 30 mM HEPES/MES become more evident when starting pH is reduced (i.e. when CO_2_/HCO_3_^−^ buffering is weaker) (Fig. 4f). Thus, it is possible to curtail medium acidification with 30 mM HEPES/MES, but less than anticipated from its buffering capacity per se. Whilst these observations should not be generalized to all cells, they emphasize the importance of making confirmatory measurements of pH, and taking into consideration the biological responses to increased buffering, such as metabolic stimulation.

Two additional precautions must be taken when using NVBs. The first issue relates to the titration of these buffers with acids (e.g. HCl) or bases (e.g. NaOH), which introduces additional osmolytes (Na^+^, Cl^−^) into the medium (Fig. 4g). The build-up of osmolytes can be substantial, for example, the addition of 20 mM HEPES and titration to pH 7.4, introduces ~30 mOsm kg^−1^ of additional osmolytes (i.e. excess of ~10%). A major increase in osmolality would lead to cell shrinkage, changes in membrane tension and potentially a myriad of downstream effects^24^. Indeed, supra-physiological osmolality is likely to influence the results of the experiment shown in Fig. 4e, f. Some media formulations are available without buffers, giving some leeway for adding extra osmolytes within physiological limits. For example, a total of 88 mOsm kg^−1^ of additional osmolytes can be added to HCO_3_^−^-free medium D7777 (Sigma-Aldrich) for the final solution to attain the same osmolality as ready-to-use medium D5796 (Sigma-Aldrich) (Fig. 4h). The second issue to consider relates to the binding properties of buffers. Although NVBs are primarily chelators of H^+^ ions, they also show modest affinity for Ca^2+^ ions. Lower [Ca^2+^] reduces the driving force for Ca^2+^ entry into cells and hence the state of Ca^2+^ signalling cascades^25^. Solutions containing 20 mM HEPES, PIPES or MES will reduce Ca^2+^ by ~10–15% (Fig. 4i). Whilst this may not have a paramount effect on Ca^2+^-dependent properties, it will contribute towards increased noise and weaker statistical power. This issue could be avoided by adding CaCl_2_ to compensate for the chelation.

### High-throughput analysis of intracellular pH

**Fig. 5:**
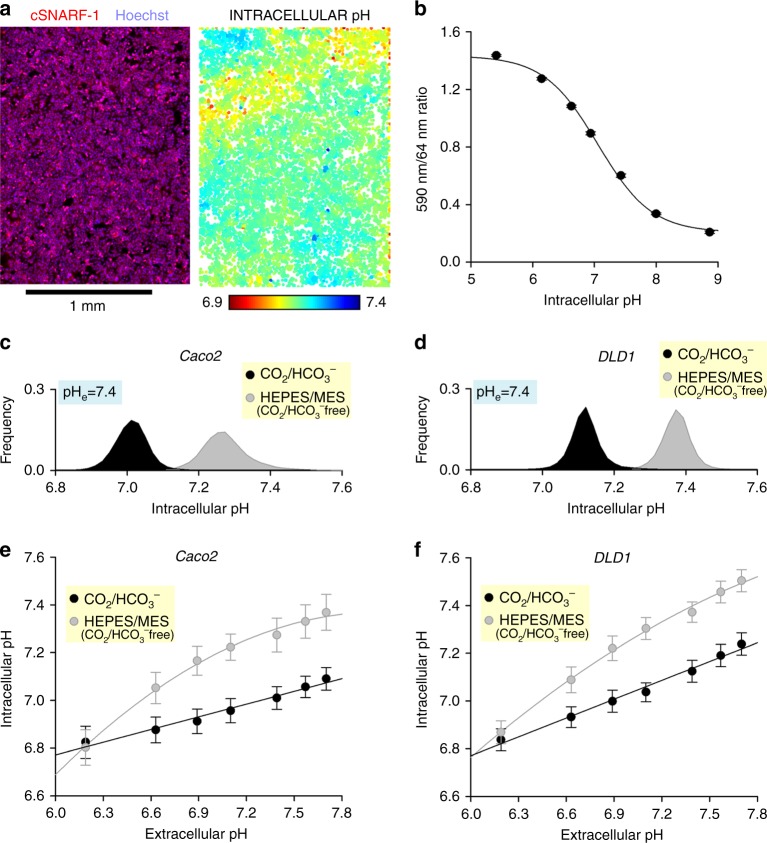
Effects of buffering regime and medium pH on intracellular pH, measured using a high-throughput imaging method. **a** Monolayer of DLD1 cells imaged with Cytation 5 plate reader. Image on left shows superimposition of cSNARF1 and Hoechst-33342 fluorescence maps. Image on right shows pH in individual cells, identified by nuclear staining. **b** Calibration curve determined from nine colorectal cancer cell lines (LS174T, PMFKO14, LS513, HCT15, SW620, GP2D, HCT116, Caco2, RW2892; seeding density 100,000 cells per well, growth area 0.56 cm^2^ per well). Three technical replicates each. Best-fit curve: pH = 6.978 + log((1.497 −ratio)/(ratio − 0.221)). **c** Histogram of intracellular pH in Caco2 monolayers bathed in D7777-based media (25 mM glucose) at pH 7.4, buffered by either 5% CO_2_/22 mM HCO_3_^−^, or 10 mM HEPES + MES titrated to 7.4 (CO_2_ free). Note the substantial alkalinization in the absence of physiological buffer. Repeated three times (three technical repeats each). **d** Experiment performed on DLD1 monolayers. **e** Effect of medium pH on intracellular pH in Caco2 monolayers. The pH of CO_2_/HCO_3_^−^-buffered media was varied by changing [HCO_3_^−^] (incubation in 5% CO_2_). In contrast, the pH of HEPES/MES-buffered media were titrated to target pH with NaOH at the bench (incubation in 0% CO_2_). Best fit: linear (CO_2_/HCO_3_^−^) or polynomial (HEPES/MES). Note that intracellular pH is more responsive to changes in extracellular pH in the absence of physiological (CO_2_/HCO_3_^−^) buffer. **f** Experiment repeated on DLD1 monolayers. Data are shown as mean ± SD. Repeated three times (three technical repeats each)

A fundamental reason why changes in medium pH (controlled or unwarranted) can influence cellular physiology is because intracellular pH (pH_i_) responds to changes in extracellular pH (pH_e_). This coupling arises because the proteins that regulate pH_i_ are also sensitive to pH_e_, and a re-balancing of transmembrane acid–base fluxes alters steady-state pH_i_. A major contributor to these acid–base fluxes are HCO_3_^−^ transporters, which are active only in the presence of CO_2_/HCO_3_^−^ buffering^8,26^. Thus, an assessment of the effects of medium pH and buffering regime on cell behaviours should consider actions mediated through changes in pH_i_. Plate-based imaging platforms allow high-throughput fluorescence measurements that can capture the population distribution of pH_i_ in a monolayer. These pH_i_ data can be obtained by loading cells with pH-sensitive fluorescence dye cSNARF1^27^. To identify the centroids of cells, nuclei can be stained with Hoechst-33342, which is spectrally resolvable from cSNARF1. After a period of loading (10 min) and wash-out in dye-free media, stacks of images were collected, corresponding to 447 nm fluorescence excited at 377 nm (Hoechst-33342) and of 590 and 640 nm fluorescence excited at 531 nm (cSNARF1) (Fig. 5a). Offline, the pH_i_ in individual cells was inferred from the cSNARF1 fluorescence probed around nuclei, identified by particle analysis of Hoechst-33342 images. After ratioing background-subtracted fluorescence at 590 and 640 nm, pH_i_ can be sampled individually for each cell (Fig. 5a). An example of a suitable code, written as a MATLAB script, is included as Supplementary Code 1. This approach was first applied to generate a calibration curve with the nigericin method^28^, in which cells are incubated in solutions containing 100 µM nigericin (a H^+^/K^+^ ionophore), 140 mM KCl (to balance intracellular K^+^), 0.5 mM EGTA, 1 mM MgCl_2_, 10 mM MES (for pH <7) or 10 mM HEPES (for pH >7) titrated to a desired target pH in a CO_2_-free atmosphere. The calibration curve shown in Fig. 5b was determined in CO_2_-free conditions, and the best-fit equation can be applied to convert measured fluorescence ratio into pH for cells under various test conditions.

This pH_i_ imaging approach was used to investigate the effect of CO_2_/HCO_3_^−^ buffering on pH_i_. Figure 5c, d show histograms of pH_i_ in populations of Caco2 or DLD1 cells, incubated either in media buffered with 5% CO_2_/22 mM HCO_3_^−^ (equilibrated at pH 7.4), or CO_2_/HCO_3_^−^-free media buffered by 10 mM HEPES and 10 mM MES (titrated to pH 7.4). In the absence of HCO_3_^−^ ions, the pH_i_ of DLD1 and Caco2 cells was shifted in the alkaline direction by 0.3 units, which is attributable to the inactivation of pH_i_-regulating HCO_3_^−^ transporters. The direction and magnitude of this effect is likely to be cell type-dependent, and therefore not intuitive to predict. By repeating these experiments over a range of media pH, it is possible to map the pH_e_–pH_i_ relationship (Fig. 5e, f). The pH_i_ of cells was more sensitive to changes in pH_e_ in the absence of CO_2_/HCO_3_^−^. For example, pH_i_ in Caco2 cells acidified by twice as much in the absence of CO_2_/HCO_3_^−^ in response to a drop in pH_e_ from 7.4 to 6.4 (Fig. 5e). Since the majority of H^+^ targets are intracellular, such buffer regime-dependent changes in pH_e_–pH_i_ coupling can lead to erroneous inferences concerning the mechanisms of cell responses to microenvironmental acid–base challenges.

## Discussion

Research in virtually every biomedical laboratory relies on cell culture, either to maintain cells in a state that is conducive for physiologically relevant activity or to explore the effects of controlled chemical, physical or biological influences. Cultured cells will remain an essential biological resource, offering a tractable model for characterizing pathways, recording responses and manipulating disease-related processes^29^. However, the translational relevance of findings borne from culture systems is critically dependent on the extent to which in vitro conditions relate to in vivo setting. Furthermore, the value of any experimental finding is determined by its reproducibility. However, 70% of scientists surveyed recently by *Nature* were unable to reproduce another’s experiment^30^, and a post-publication analysis has suggested a reproducibility rate of as little as 10% in cancer biology^31^. The inadequate quality of preclinical data has been linked to the high failure rate of agents progressing from in vitro validation to phase III testing, of the order of 95% in cancer research^32^. One factor contributing towards these outcomes has been attributed to variables relating to environmental conditions^31^.

**Fig. 6:**
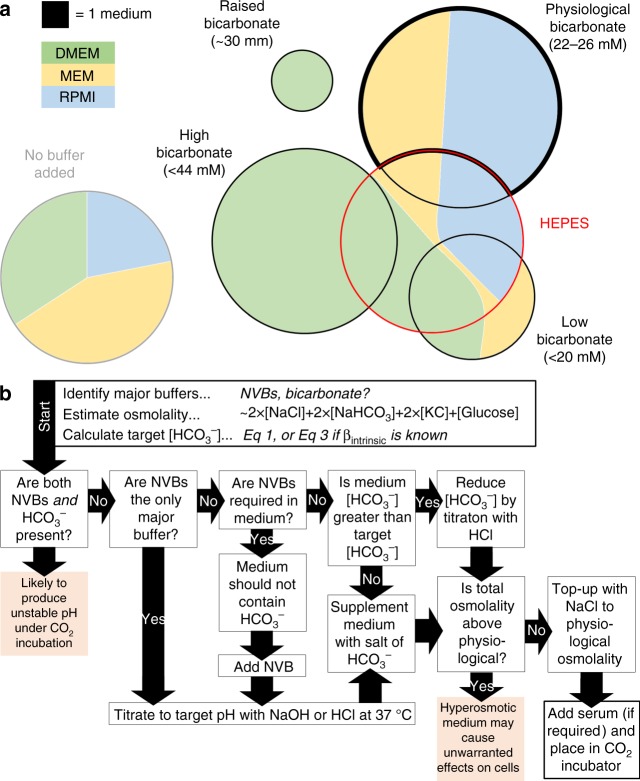
Summary of buffering regimes in commercially-available media formulations, and flow chart showing instructions for preparing media at a target pH. **a** Venn diagrams summarizing the commercial availability of Dulbecco’s modified Eagle’s medium (DMEM), minimum essential medium (MEM) or RPMI-1640 buffers (supplied by Sigma-Aldrich and Thermo Fisher Scientific), grouped by buffering regime. Area is proportional to the number of media available in each category. Media with physiological HCO_3_^−^ and no additional non-volatile buffer are highlighted with a thick black border. HEPES-buffered media are indicated with a red outline. **b** Flow chart guiding through the steps required to adjust the pH of culture media. NVB: non-volatile buffer (e.g. HEPES). Target [HCO_3_^−^] for a given medium pH can be calculated from Eq. 3. Total osmolality can be approximated as 2 × [NaCl] + 2 × [NaHCO_3_] + 2 × [KCl] + [Glucose]. See Supplementary Data 1 for further details of these steps

Commercial sources of media offer a wide range of formulations, summarized in Fig. 6a for three major types: DMEM, MEM and RPMI-1640. Fewer than half of the available formulations contain physiological [HCO_3_^−^], and a substantial number of options include media with considerably lower or higher [HCO_3_^−^], which would produce acidic and alkaline conditions, respectively (Fig. 2a). Special precautions are needed with various formulations supplemented with HEPES because these may produce unexpected pH dynamics inside CO_2_ incubators (Fig. 3). Formulations that lack CO_2_/HCO_3_^−^ and any major NVB provide are useful starting point for producing bespoke media (Fig. 4).

In a retrospective review of articles published in *Nature* and *Cancer Research* (third quartile of 2017, a period selected at random for the purpose of this analysis) reveals that only a small percentage of studies provide the necessary information about the buffering regime and pH of culture media. Three-quarters of articles published in *Cancer Research* and two-thirds of life science articles published in *Nature* present data from cultured cells. However, just under half of these articles report the manufacturer of the medium, and only a tenth give information about the buffer composition. Only a third of all studies report the pCO_2_ in incubators: typically 5%, although some using 10% CO_2_ (which then necessitates a proportional adjustment to HCO_3_^−^). A significant number of studies use media containing [HCO_3_^−^] outside the range 22–26 mM, producing a non-physiological pH. Among the studies that reported the use of 5% CO_2_, approximately one-third used classical DMEM, the underlying formulation of which contains 44 mM HCO_3_^−^ (which would equilibrate to pH 7.7 in 5% CO_2_). Less than a tenth of studies used media supplemented with HEPES, half of which were a mixture of HEPES and bicarbonate, which are identified herein as potentially problematic (Fig. 3).

Attaining a fine degree of control over pH is realistically achievable in modern culture systems, and efforts should be made to implement the best practice in a bid to improve the accuracy, compatibility and reproducibility of measurements. The flow chart in Fig. 6b illustrates the suggested steps in setting the pH of media. Supplementary Data 1 provides further details of these steps, illustrated in a selection of media from a major supplier. Based on the observations described herein, we make the following recommendations:

**Recommendation 1: CO**_**2**_**/HCO**_**3**_^**−**^ **is the physiological buffer and therefore should be the preferred choice for biological research.** Its use avoids possible unwarranted effects that exogenous buffers may have^19^, for example, longer-term toxicity^33–35^, Ca^2+^ binding (Fig. 4i) or glycolytic stimulation (Fig. 4c–f). Including CO_2_ as part of the buffering regime also establishes a more realistic transmembrane [CO_2_] gradient for those cells that generate CO_2_. Additionally, the presence of HCO_3_^−^ ions activates essential membrane transport processes responsible for cellular pH homeostasis^8,26^. This influences steady-state intracellular pH and its sensitivity to changes in extracellular pH_e_ (Fig. 5c–f), an important transduction mechanism by which medium pH modulates cellular behaviours.

**Recommendation 2: media exposed to an atmosphere enriched in CO**_**2**_ **must include an appropriate concentration of HCO**_**3**_^**−**^ **salt in order to stabilize at the required pH.** CO_2_/HCO_3_^−^ is unusual among buffers because its acidic component is a gas. Consequently, precautionary measures are warranted when handling CO_2_/HCO_3_^−^-buffered media in open chambers to avoid the loss of gas, and hence alkalinization. Whilst this chemical peculiarity is desirable in vivo because it allows the lungs to regulate buffering, it poses a challenge for experiments involving changes in ambient pCO_2_. Media that are to be exposed to a raised pCO_2_ (e.g. inside a CO_2_ incubator) must contain a salt of HCO_3_^−^ in order for the buffer to promptly stabilize at a predictable pH. Conveniently, medium pH could be set by changing the ratio of pCO_2_ to [HCO_3_^−^] (Fig. 1c). When taking readings outside CO_2_ incubators, it is important to consider the pH dynamics associated with abrupt shifts in pCO_2_. The pH of media in small volumes (e.g. 200  µL) will begin to rise immediately when removed from a CO_2_ incubator, and may require hours to attain the new equilibrium. Conversely, when preparing media for incubation, adequate time should be allowed for equilibration inside a CO_2_ incubator. This mitigates the risk of an unwarranted transient alkaline stimulus imposed on cells by an out-of-equilibrium medium.

**Recommendation 3: media supplemented with non-volatile buffers  may have unstable pH if these are prepared without consideration of the CO**_**2**_**-HCO**_**3**_^**−**^ **equilibrium.** For periods outside CO_2_ incubators, non-volatile buffers can be used to stabilize pH, provided that HCO_3_^−^ salts are not included (to match the absence of CO_2_). Conversely, for experiments involving CO_2_ incubation, non-volatile buffers should not be used in lieu of HCO_3_^−^, as this results in media becoming more acidic than anticipated. If there is a good biological reason to supplement physiological CO_2_/HCO_3_^−^ with a non-volatile buffers, the medium should first be prepared with the non-volatile buffers, titrated to the target pH, and then supplemented with a combination of CO_2_ and HCO_3_^−^ that is expected to be at equilibrium with the target pH. Some ready-made media, containing mixtures of several buffers, may not be compatible with this sequence, and thus yield unstable pH dynamics under CO_2_ incubation. Additionally, when preparing bespoke media with non-volatile buffers, changes in free [Ca^2+^] and total osmolality must be considered to avoid non-physiological conditions.

**Recommendation 4: reporting standards must provide adequate information about the buffering regime.** This should include a description of buffer composition, CO_2_ partial pressure and, in the case of bespoke media, the steps involved in preparing media.

## Methods

### Cell lines and culture conditions

Human colorectal adenocarcinoma cells Caco2, DLD1 and NCI-H747 cells were obtained from Prof. Walter Bodmer’s collection at the Weatherall Institute of Molecular Medicine (University of Oxford, UK). Caco2 and DLD1 cells were cultivated in DMEM (Life technologies, Cat. No. 41965-039) supplemented with 10% FBS (Sigma-Aldrich) and 1% PS (100 U mL^−1^ penicillin, 100 µg mL^−1^ streptomycin; Sigma-Aldrich). NCI-H747 cells were cultivated in RPMI-1640 (Thermo Fisher Scientific, Cat. No. 21875-034), in 5% CO_2_ and at 37 °C. Alternatively, cells were treated with media based on NaHCO_3_-free DMEM (Sigma-Aldrich, Cat. No. D7777), supplemented with various concentrations of NaHCO_3_, NaCl, HEPES, PIPES or MES, as indicated in figure legends or NaHCO_3_ and glucose-free DMEM (Sigma-Aldrich, Cat. No. D5030) supplemented with various concentrations of glucose and NaCl, as indicated in figure legends. Lines were authenticated by  single nucleotide polymorphism (SNP)-based profiling and tested routinely for mycoplasma contamination.

### Monitoring medium pH using absorbance

Medium pH was measured by PhR absorbance at 430 and 560 nm using Cytation 5 imaging plate reader (Biotek) equipped with a CO_2_ gas controller (Biotek). Measurements were taken from 200 µL medium in clear, flat-bottom 96-well plates (Costar) with lids at 37 °C. Media were based on NaHCO_3_-free DMEM (Sigma-Aldrich, Cat. No. D7777), supplemented with 10% FBS, 1% PS and various concentrations of NaHCO_3_, NaCl, HEPES, PIPES or MES, as indicated in figure legends. Alternatively, media based on NaHCO_3_-free, glucose-free DMEM (Sigma-Aldrich Cat. No. D5030) supplemented with 10% FBS, 1% PS and various concentrations of glucose and NaCl, as indicated in figure legends was used.

### Cell growth analysis using SRB

Cells were plated in triplicates at densities of 4,000 cells per well in clear, flat-bottom, 96-well plates with a growth area of 0.32 cm^2^ per well (Costar). The following day, the medium was replaced with 200 µL DMEM (Sigma-Aldrich, Cat. No. D7777), supplemented with  10% FBS, 1% PS and various concentrations of NaHCO_3_, NaCl, HEPES, PIPES or MES, as indicated in figure legends. Alternatively, media based on NaHCO_3_-free, glucose-free DMEM (Sigma-Aldrich Cat. No. D5030) supplemented with 10% FBS, 1% PS and various concentrations of glucose and NaCl, as indicated in figure legends was used. Cells were cultured for 6 days and pH_e_ was monitored on each day using PhR absorbance. After 6 days, the cells were fixed using 10% trichloroacetic acid at 4 °C for 60 min. Afterwards, they were washed with H_2_O four times, and stained with SRB (0.057% in 1% acetic acid) for 30 min. Residual SRB was removed by washing four times with 1% acetic acid. SRB was then dissolved in 10 mM Tris base. SRB absorbance was read at 520 nm absorbance using Cytation 5 imaging plate reader (Biotek).

### pH_i_ measurements

Cells were plated in triplicate at 100,000 cells per well in black wall, flat coverslip bottom µ-plate 96-well plates with a growth area of 0.56 cm^2^ per well (Ibidi) and were left to attach overnight. They were then incubated in media supplemented with cSNARF1-AM (5 µg mL^−1^, Molecular Probes) and the nuclear stain Hoechst-33342 (10 µg mL^−1^, Molecular Probes), for 10 min, and then replaced with dye-free medium (twice). Images of fluorescence excited at 377 nm and collected at 447 nm (Hoechst-33342), and of fluorescence excited at 531 nm and collected at 590 nm and 640 nm (cSNARF1), were acquired using Cytation 5 imaging plate reader and its bespoke software. For media buffered with CO_2_/HCO_3_^−^, measurements were performed in an atmosphere of 5% CO_2_, established in the plate reader. Further analysis of the population distribution of pH data was performed with a MATLAB script (Supplementary Code 1).

### Lactate and free calcium measurements

Free lactate and calcium concentrations were determined using ABX Pentra 400 (Horiba) from 150 µL aliquots of medium.

### Reporting summary

Further information on experimental design is available in the Nature Research Reporting Summary linked to this article.

## Supplementary information

Supplementary Information

Description of Additional Supplementary Files

Supplementary Data 1

Supplementary Data 2

Supplementary Code 1

Reporting Summary

## Data Availability

The datasets generating and analysed in this study are available for download as Supplementary Data 2.
