# Supplementary material for: Evidence-based guidelines for controlling pH in mammalian live-cell culture systems
Source: Commun Biol. 2019 Apr 26;2:144. doi: 10.1038/s42003-019-0393-7 (PMC6486606; doi:10.1038/s42003-019-0393-7)
Supplement: Supplementary file 1 — Supplementary Information [file 42003_2019_393_MOESM1_ESM.pdf]

SUPPLEMENTARY NOTE

Deriving buffering capacity

In any biological system, a large pool of H^+ ions is held on buffers. This is represented chemically by the equilibrium involving the buffer's ionised (B) and protonated (HB) states:

$$\mathrm{B} + \mathrm{H}^+ = \mathrm{HB}$$

The acid-dissociation constant of this equilibrium is defined as:

$$K_a = \frac{[\mathrm{H}^+]\cdot[\mathrm{B}]}{[\mathrm{HB}]} \quad \text{(Eq 4)}$$

The system's buffering capacity can be expressed as the ratio between the change in [B] (or [HB]) and the measured [H^+] response. In a closed system, the sum of [B] and [HB] is constant (C_B). By applying this to Eq 4, it is possible to derive an expression for [B]:

$$[\mathrm{H}^+] = K_a \cdot \frac{C_B - [\mathrm{B}]}{[\mathrm{B}]}$$

$$[\mathrm{B}] = \frac{K_a \cdot C_B}{K_a + [\mathrm{H}^+]}$$

Buffering capacity, quantified as Δ[B]/Δ[H^+], becomes a derivative for infinitesimal [H^+] changes:

$$\frac{d[\mathrm{B}]}{d[\mathrm{H}^+]} = -\frac{K_a \cdot C_B}{(K_a + [\mathrm{H}^+])^2}$$

Since pH is the reporting standard for changes in [H^+], a more appropriate quantification for buffering is *d*[B]/*d*pH, which can be expanded by a mathematical formula called the chain rule:

$$\frac{d[\mathrm{B}]}{\mathrm{dpH}} = \frac{d[\mathrm{B}]}{d[\mathrm{H}^+]}\cdot\frac{d[\mathrm{H}^+]}{dpH} = \left(-\frac{K_a \cdot C_B}{(K_a + [\mathrm{H}^+])^2}\right)\cdot(-\ln(10)\cdot[\mathrm{H}^+])$$
$$= \ln(10)\cdot C_B \cdot \frac{K_a \cdot [\mathrm{H}^+]}{(K_a + [\mathrm{H}^+])^2} = \ln(10)\cdot C_B \cdot \frac{10^{pK_a - pH}}{(1 + 10^{pK_a - pH})^2} \quad \text{(Eq 5)}$$

Note that *d*pH/*d*[H^+] is the first derivative of $-\log_{10}([H^+])$. Eq 5 shows that peak buffering capacity is attained when medium pH aligns with the buffer's pK_a. Under these circumstances, the buffer's protonated and unprotonated forms are equal in concentration (Eq 1).

In contrast to closed buffer systems, CO_2/HCO_3^- is considered an open system because its components can be biologically regulated. For media inside a CO_2 incubator, the concentration of the acidic form of the buffer (rather than the sum of the acidic and basic forms) is constant. Adapting this to Eq 4,

$$K_a = \frac{[\mathrm{H}^+]\cdot[\mathrm{HCO_3^-}]}{[\mathrm{CO_2}]}$$

$$[\mathrm{HCO_3^-}] = \frac{K_a \cdot [\mathrm{CO_2}]}{[\mathrm{H}^+]}$$

$$\frac{d[\mathrm{HCO_3^-}]}{d[\mathrm{H}^+]} = -\frac{K_a \cdot [\mathrm{CO_2}]}{([\mathrm{H}^+])^2}$$

$$\frac{d[\mathrm{HCO_3^-}]}{d\mathrm{pH}} = \ln(10)\cdot\frac{K_a \cdot [\mathrm{CO_2}]}{[\mathrm{H}^+]} = \ln(10)\cdot[\mathrm{HCO_3^-}]$$

Henderson-Hasselbalch correction

According to the Henderson-Hasselbalch equation, the concentration of HCO_3^- required to attain a given target pH (pH_{target}) at 5% CO_2 (1.2 mM) is:

$$[\mathrm{HCO_3}^-] = 1.2 \times 10^{\mathrm{pH_{target}} - 6.15}$$

When a medium containing a salt of HCO_3^- and intrinsic buffers (e.g. serum proteins) is placed in a 5% CO_2 incubator, the CO_2 hydration reaction proceeds until equilibrium is reached:

$$CO_2 \leftrightarrow HCO_3^- + H^+$$

Intrinsic buffers will chelate some of the H^+ ions, allowing the reaction to proceed towards a combination of higher [HCO_3^-] and lower [H^+]. Consequently, the standard Henderson-Hasselbalch equation will underestimate the pH of solutions prepared with low [HCO_3^-]. The necessary correction can be derived mathematically by solving a system of differential equations:

$$\frac{d\mathrm{pH}}{dt} = -\frac{k_h \cdot [\mathrm{CO_2}] - k_r \cdot [\mathrm{HCO_3}^-] \cdot [\mathrm{H}^+]}{\beta_{intrinsic}}$$

$$\frac{d[\mathrm{HCO_3}^-]}{dt} = k_h \cdot [\mathrm{CO_2}] - k_r \cdot [\mathrm{HCO_3}^-] \cdot [\mathrm{H}^+]$$

Here, k_h is the CO_2 hydration constant (0.19 s^{-1}), k_r is the rate constant of the reverse reaction (equal to K_a/k_h) and [CO_2] takes a constant value of 1.2 mM in a 5% CO_2 incubator. Intrinsic buffering capacity is given by a constant $\beta_{intrinsic}$. The equation can be solved for a given starting [HCO_3^-] to obtain a steady-state relationship (i.e. $d\mathrm{pH}/dt = d[\mathrm{HCO_3^-}]/dt = 0$) between added [$HCO_3^-$] and pH at 5% CO_2. Assuming serum has a pH of 7.4, it can be shown that the correction to starting [HCO_3^-] is:

$$\Delta[\mathrm{HCO_3}^-] = \beta_{intrinsic} \times (\mathrm{pH_{target}} - 7.4)$$

Thus, [HCO_3^-] required to produce a medium at pH_{target} in 5% CO_2 is given by:

$$[\mathrm{HCO_3}^-] = 1.2 \times 10^{\mathrm{pH_{target}} - 6.15} + \beta_{intrinsic} \times (\mathrm{pH_{target}} - 7.4) \quad \text{(Eq 6)}$$

Sera used in this study produced a $\beta_{intrinsic}$ of 1.11 mM pH^{-1} (**Fig 1C**), determined by best-fit, but will likely vary depending on the type and concentration of serum used.
