# Supplementary material for: Evidence-based guidelines for controlling pH in mammalian live-cell culture systems
Source: Commun Biol. 2019 Apr 26;2:144. doi: 10.1038/s42003-019-0393-7 (PMC6486606; doi:10.1038/s42003-019-0393-7)
Supplement: Supplementary file 2 — Description of Additional Supplementary Files [file 42003_2019_393_MOESM2_ESM.docx]

**Description of Additional Supplementary Files**

**File Name**: Supplementary Data 1

**Description:** Details of the steps for setting the pH of media

**File Name**: Supplementary Data 2

**Description**: Datasets presented in the Figures

**File Name**: Supplementary Code 1

**Description**: Example of algorithm written as a MATLAB m script for analysing population statistics on intracellular pH
